# Long‐term seizure and developmental outcomes of epilepsy surgery in children under 3 years old: A single‐center study of 113 patients

**DOI:** 10.1111/cns.14481

**Published:** 2023-10-03

**Authors:** Hao Yu, Qingzhu Liu, Ruofan Wang, Chang Liu, Yu Sun, Yao Wang, Taoyun Ji, Shuang Wang, Xiaoyan Liu, Yuwu Jiang, Lixin Cai

**Affiliations:** ^1^ Pediatric Epilepsy Center Peking University First Hospital Beijing China

**Keywords:** children, development, disconnection, drug‐resistant epilepsy, surgery

## Abstract

**Aims:**

To investigate the clinical characteristics, surgical strategy, developmental and seizure outcomes, and predictors of surgical outcome in children with drug‐resistant epilepsy (DRE) under 3 years old.

**Methods:**

One hundred thirteen consecutive children younger than 3 years of age with DRE underwent curative surgical treatment after multidisciplinary preoperative evaluation using the strategy developed in the pediatric epilepsy center of Peking University First Hospital (PKFHPEC) between 2014 and 2018. These patients were selected for retrospective study. The relevant clinical data were collected and analyzed. The surgical prognoses were classified using the Engel classification, and the developmental assessment results were collected. Statistical analysis of the clinical data was performed to analyze the predictors of seizure outcomes and their correlation with developmental outcomes.

**Results:**

All the patients were followed up for more than 3 years, and 98 (86.7%) patients had no seizure recurrence. One year after surgery, the seizure‐free rate was 86.7%, which was as high as that at the last follow‐up. Cortical dysplasia was the most frequent etiology of DRE in this cohort, accounting for 77.0%. According to the Engel classification, acute postoperative seizure (APOS; *p* < 0.001) was a predictor of seizure recurrence. No deaths occurred. No unpredicted long‐term severe complications occurred except for one ventricular peritoneal shunt. The patients' neurodevelopmental statuses were improved after successful surgery, while the scores of the pre‐ and postoperative developmental assessments were closely correlated.

**Conclusions:**

For children who are younger than 3 years old and have DRE and structural abnormalities, early curative treatment can lead to long‐term good seizure outcomes and a low complication rate. The development of appropriate strategies for both presurgical evaluation and resection is crucial for the success of surgery.

## Graphical abstract

86.7% patients had no seizure recurrence in our drug‐resistant epilepsy cohort. Acute postoperative seizure was a predictor of seizure recurrence. Early curative treatment can improve neurodevelopmental statuses. Appropriate strategies for both presurgical evaluation and resection are crucial for the success of surgery.

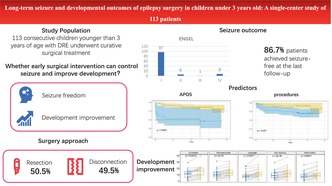

## 1 INTRODUCTION

The most common chronic neurologic disorder in children is epilepsy. The incidence of epilepsy is highest in children during the first few years of life.^1^, ^2^, ^3^ In a multicenter survey about pediatric epilepsy surgery that was proposed by the International League Against Epilepsy (ILAE), 46% of patients had their first seizure before turning 1 year old.^4^ Up to 30% of patients have seizures that fail to respond to at least two antiseizure medicines (ASMs); such cases are characterized as drug‐resistant epilepsy (DRE).^5^ DRE can be efficiently controlled by timely epileptic surgery, which is well documented as safe and successful^6^, ^7^, ^8^ and eventually prevents cognitive and psychosocial decline that is often induced by epilepsy.^9^, ^10^ Moreover, the younger the patient at the time of surgery, the more complete the excision because of cerebral plasticity, thereby reducing the side effects of ASMs.^11^

However, epilepsy surgery is rarely performed in infants because of high surgical risks, such as low blood volume, fragile vessels, and complicated evaluation of the epileptogenic zone (EZ) due to an immature network of the nervous system and epileptic encephalopathy. There have been relatively few studies investigating the effects of DRE surgery on children under 3 years of age, and most of them had small cohorts.^12^ Multicenter cross‐sectional studies had large sample sizes but had different strategies for conducting the presurgical evaluation and had a long investigation period, thereby convoluting the conclusion.^13^

In this study, we collected the clinical data of patients who were treated in a single pediatric epilepsy center and followed up for more than 3 years postoperatively. We defined the clinical characteristics, diagnostic modalities, rationale of presurgical evaluation, surgical techniques, and complications as well as the prognosis to explore the potential predictors of seizure outcomes in children under 3 years of age.

## 2 MATERIALS AND METHODS

### 2.1 Inclusion criteria of patients

From August 2014 to March 2018, a consecutive series of 439 children with DRE were admitted to the pediatric epilepsy center of Peking University First Hospital (PKFHPEC) for curative surgical treatment after multidisciplinary preoperative evaluation. Inclusion criteria: (a) patients who were diagnosed with DRE according to the criteria defined by the ILAE^14^ or patients who had frequent seizure attacks and showed structural epileptogenic lesions on MRI despite having a short epilepsy duration; (b) patients who were younger than 3 years of age at the time of surgery; and (c) patients who were followed up for more than 3 years postoperatively.

### 2.2 VEEG, MRI, and PET‐CT examinations

All the selected children underwent presurgical evaluation, including long‐term scalp video electroencephalogram (VEEG) using the standard 10–20 system, which recorded usual convulsions at least 3 times, and 3D brain high‐resolution magnetic resonance imaging (MRI; 3.0 T), including T1, T2, FLAIR, and DWI sequences, and fluorodeoxyglucose positron emission tomography (FDG‐PET) brain examination. MRI‐PET were registered with Sinovision software (Beijing Sinovision Medical Technology Co., Ltd.), thereby enabling MRI and PET‐CT to be combined for a more intuitive 3D reconstruction. All these factors made surgical planning easier. No patient in this cohort underwent intracranial EEG.

### 2.3 Surgery

After craniotomy, EZs were identified by electrocorticography (ECoG). Motor evoked potentials (MEPs) and somatosensory evoked potentials (SEPs) were performed in cases of lesions in the central cortex. Surgical types included resection and disconnection procedures. A few patients underwent both procedures if needed. All the disconnection procedures were followed by partial resection of brain tissue, with the aim of collecting specimens for pathological analysis and exposing key points about the lesion anatomy to contribute to the next disconnection step. Peri‐insular hemispherotomy (PIH)^15^ was performed for all patients with hemispheric lesions. The extent of surgery was cataloged as (a) hemispheric; (b) within one lobe (including lobar lesion); and (c) multiple lobar. All surgeries were performed by the same senior neurosurgeon with his surgery team.

### 2.4 Seizure outcome

The seizure outcomes were evaluated using the Engel classification.^16^ If the patients underwent a reoperation, only the final follow‐up results were used regardless of whether it was the second or third surgery. The occurrence of an APOS within the first postoperative week was not identified as seizure recurrence.^17^ If seizures recurred, the first recurrent seizure after surgery was regarded as the onset of seizure recurrence in the Kaplan–Meier survival analysis. Generally, VEEG was conducted after surgery, and the last ASM was discontinued at least 2 years after surgery.

### 2.5 Developmental assessment

The Griffiths Mental Development Scales‐China (GMDS‐C) was used for developmental assessment. GMDS‐C was performed before surgery, and within 1 month and at 3 months after surgery. Raw scores and developmental quotients (DQs) before and after surgery in each domain of the scale were compared.

### 2.6 Ethics and informed consent

This study was approved by the Peking University First Hospital ethics committee. All the participants' parents gave their written informed consent regarding the use of their children's data for research.

### 2.7 Statistical analysis

The statistical software used was R 4.2.2 (Lucent Technologies). The Shapiro–Wilk test was used as the formal test for normality. The statistical method for prognostic factor analysis was *t*‐test and the nonparametric independent‐sample Kruskal–Wallis test. The paired *t*‐test was the statistical method for comparative analysis of pre‐ and postoperative developmental assessment. Pearson's test was used for correlation analysis of developmental assessment results. Variables with a significance level <0.05 in the initial univariate analysis were then tested in multivariate Cox analysis. A Kaplan–Meier survival curve was utilized to estimate the probability of seizure freedom over time.

## 3 RESULTS

### 3.1 Patient clinical features

A total of 113 patients with ages ranging from 5.76 to 35.88 months (median 19.08 months) at the time of surgery were admitted, including 72 males. Age at seizure onset ranged from 0.01 to 30 months, with a median of 3.00 months. The epilepsy duration ranged from 3.00 to 33.48 months (median 18.12 months), and 35 patients had a course of disease that was <12 months.

A total of 12 patients had a definite family history. A total of 33 children underwent preoperative genetic whole‐exome testing, and 4 of them had pathogenic genes related to epilepsy, including CACNA1A, CREBBP, MTOR, and NPRL2 (Table 1).

**TABLE 1:** Demographic characteristics of 113 patients at baseline.

Characteristics	Overall, *N* = 113
Surgery age (months)	19.08 (5.76–35.88)
Duration (months)	18.12 (3.00–33.48)
Onset age (months)	3.00 (0.01–30.00)
Follow‐up time (years)	5.76 (4.59–8.16)
Sex	
Male	72 (63.7%)
Female	41 (36.3%)
Gene (total = 33)	
Negative	29 (87.9%)
Positive	4 (12.1%)
Family history	
Yes	12 (10.6%)
No	101 (89.4%)
Number of seizure types	
1	48 (42.5%)
2	53 (46.9%)
3	10 (8.8%)
4	2 (1.8%)
Semiology	
Focal	31 (27.4%)
Spasms	18 (15.9%)
Spasms + focal	45 (39.8%)
Multifocal	19 (16.9%)
Frequency	
>10 per day	36 (31.9%)
<10 per day	74 (65.5%)
Per week	3 (2.6%)
Lesion side	
L	66 (58.4%)
R	47 (41.6%)

*Note*: Median (range); *n* (%).

### 3.2 Presurgical examinations

One hundred eight (95.6%) children suffered seizures every day. Forty‐one patients experienced more than one seizure type. Overall, epileptic spasm was the most common type, affecting 60.2% of all the patients. Twenty‐seven percent of patients had only one type of semiology, not spasms, that corresponded with focal seizures.

Interictal EEG showed that 57.5% of patients had focal epileptic discharges, 16.8% had multifocal lesions, 10.6% had multifocal/generalized, 1.8% had burst suppression, and 13.3% had hypsarrhythmia. In the ictal EEG, focal onset on EEG accounted for 80.5% and generalized/multiple‐focal onset on EEG accounted for 19.5%. All patients had structural lesions on MRI images, 38.1% of lesions were restricted to one lobe, 28.3% to multiple lobes and 33.6% to the hemisphere. Excluding 6 who did not undergo PET‐CT, 103 had hypometabolism and 4 had hypermetabolism on PET‐CT images (Table 2).

**TABLE 2:** Clinic characteristics univariate analysis for seizure outcome.

Characteristics	Overall, *N* = 113 (%)^a^	Engel		*p* Value^b^
		I, *N* = 98 (%)^a^	II–IV, *N* = 15 (%)^a^	
Interictal EEG				
Focal	65 (57.5)	58 (59.2)	7 (46.6)	0.374
Multifocal	19 (16.8)	16 (19.4)	3 (20.0)	
Multifocal generalized	12 (10.6)	11 (11.2)	1 (6.7)	
Burst suppression	2 (1.8)	1 (1.0)	1 (6.7)	
Hypsarrhythmia	15 (13.3)	12 (12.2)	3 (20.0)	
Onset EEG				
Focal	91 (80.5)	80 (81.6)	11 (73.3)	0.487
Generalized/multiple‐focal	22 (19.5)	18 (18.4)	4 (26.7)	
Spasms				
Yes	38 (33.6)	32 (32.7)	6 (40.0)	0.575
No	75 (66.4)	66 (67.3)	9 (60.0)	
MRI abnormality extension				
One lobe	43 (38.1)	41 (41.8)	2 (13.3)	0.483
Multilobe	32 (28.3)	25 (25.5)	7 (4.7)	
Hemisphere	38 (33.6)	32 (32.7)	6 (4.0)	
MRI abnormality comparing to res/dis				
Smaller than res/dis	9 (8.0)	8 (8.6)	1 (6.7)	<0.001
Equal to res/dis	92 (81.4)	86 (87.8)	6 (40.0)	
Larger than res/dis	12 (10.6)	4 (4.1)	8 (53.3)	
PET metabolism (total = 107)				
Hypometabolism	103 (96.3)	90 (96.8)	13 (92.9)	0.434
Hypermetabolism	4 (3.7)	3 (3.2)	1 (7.1)	
PET abnormality extension (total = 107)				
Smaller than res/dis	14 (13.1)	12 (12.9)	2 (14.3)	0.120
Equal to res/dis	48 (44.9)	45 (48.4)	3 (21.4)	
Larger than res/dis	45 (42.0)	36 (38.7)	9 (64.3)	
Surgery procedure				
Resection	57 (50.4)	55 (56.1)	2 (13.3)	0.002
Disconnection	56 (49.6)	43 (43.9)	13 (86.7)	
Surgery region				
Hemisphere	35 (31.0)	29 (29.6)	6 (40.0)	0.139
Multi‐lobes	32 (28.3)	25 (25.5)	7 (46.6)	
Frontal	20 (17.7)	19 (19.4)	1 (6.7)	
Temporal	18 (15.9)	18 (18.4)	0 (0.0)	
Parietal	8 (7.1)	7 (7.1)	1 (6.7)	
MEP/SEP				
No	78 (69.0)	69 (70.4)	9 (60.0)	
Yes	35 (31.0)	29 (29.6)	6 (40.0)	
Pathology				
MCD	87 (77.0)	74 (75.5)	13 (86.7)	0.678
Encephalomalacia	10 (8.8)	8 (8.2)	2 (13.3)	
SWS	1 (0.9)	1 (1.0)	0 (0.0)	
Tumor	11 (9.7)	11 (11.3)	0 (0.0)	
TSC	2 (1.8)	2 (2.0)	0 (0.0)	
Encephalitis	2 (1.8)	2 (2.0)	0 (0.0)	
APOS				
Yes	15 (13.3)	7 (7.1)	8 (53.3)	<0.001
No	98 (86.7)	91 (92.9)	7 (46.7)	
Complication				
None	111 (98.2)	97 (99.0)	14 (93.3)	0.249
Hydrocephalus	2 (1.8)	1 (1.0)	1 (6.7)	

Abbreviations: APOS, acute postoperative seizure; Dis, disconnection; EEG, electroencephalogram; MCD, malformation of cortical dysplasia; MEP, motor evoked potentials; MRI, magnetic resonance imaging; PET, positron emission tomography; Res, resection; SEP, somatosensory evoked potentials; SWS, Sturge–Weber syndrome; TSC, tuberous sclerosis complex.

^a^ *n* (%).

^b^ Wilcoxon rank sum test; Fisher's exact test; Pearson's chi‐squared test.

### 3.3 Surgical procedures and pathology

**FIGURE 1:**
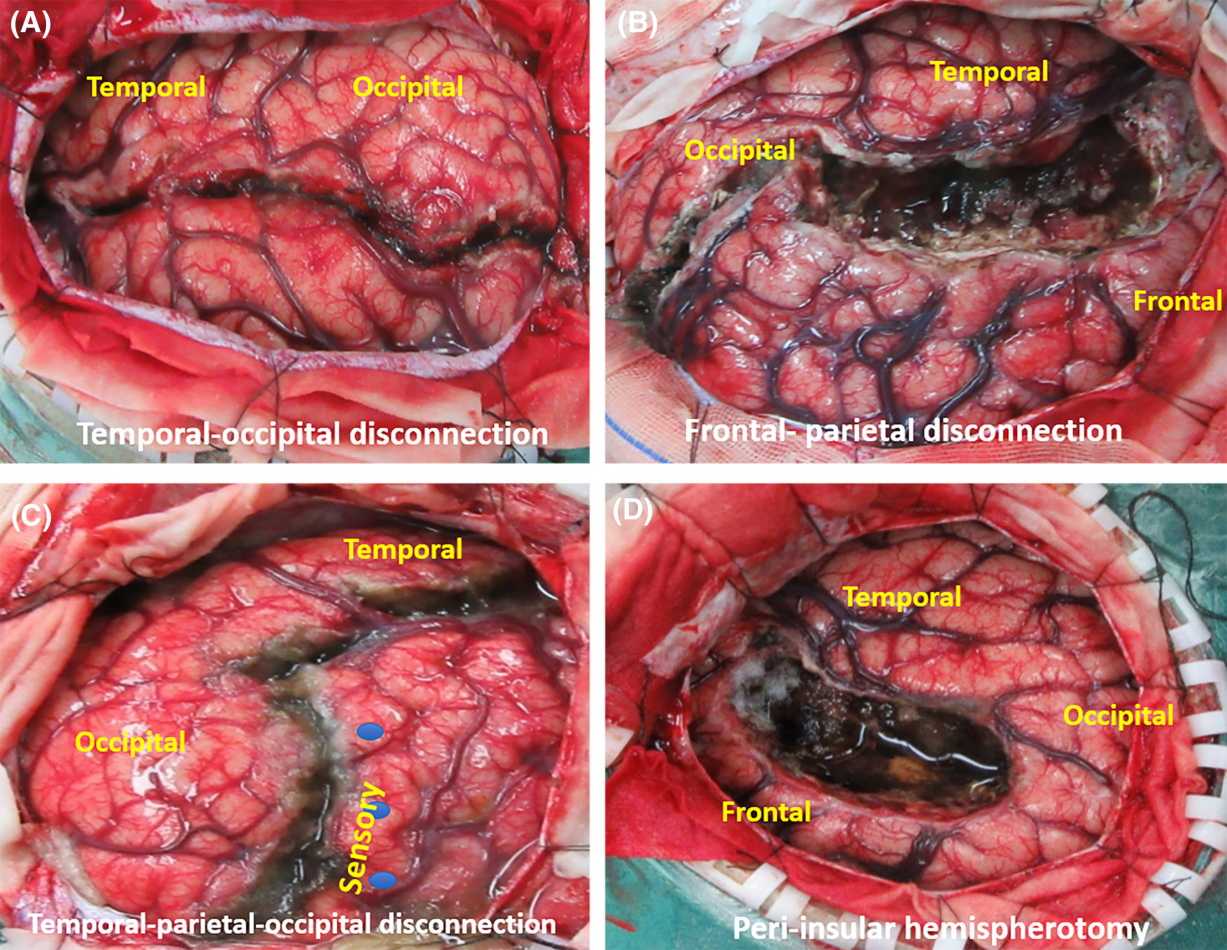
Approaches of disconnection surgery illustration. (A) Temporal–occipital disconnection surgery. (B) Frontal–parietal disconnection surgery. (C) Temporal–parietal–occipital disconnection surgery. (D) Peri‐insular hemispherotomy surgery.

**FIGURE 2:**
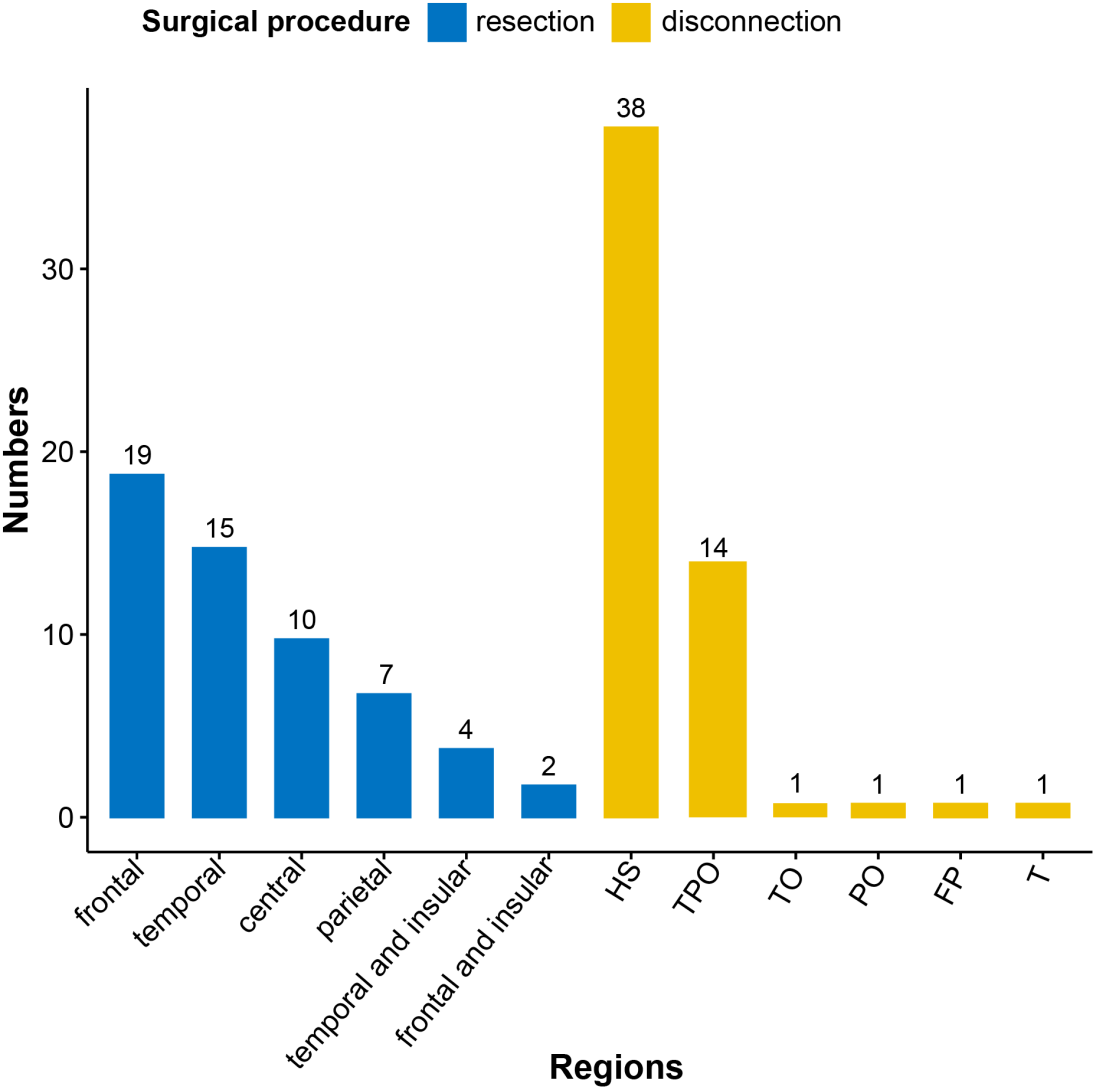
Peri‐insular hemispherotomy surgery was chosen for patients with a hemisphere lesion. In the figure, the words in capital letters indicate disconnection surgery. FP, frontal–parietal disconnection; HS, hemispherotomy; PO, parietal–occipital disconnection; T, temporal disconnection; TO, temporal‐occipital disconnection; TPO, temporal–parietal–occipital disconnection.

Fifty‐seven (50.4%) patients underwent resection, while 56 others (49.6%) underwent disconnection surgeries, which had different types (Figure 1) and surgical approaches (Figure 2). Sixty‐seven children underwent multiple lobectomies and hemispherotomies. Fifteen patients experienced surgical failure at the first attempt and underwent more than one curative surgery or palliative surgery.

Ninety‐two patients had abnormalities on MRI that were the same size as the planned resection or disconnection area; however, the extent of surgery was smaller in nine cases and larger in 12 cases. In addition to 14 cases in which the abnormalities on PET were smaller and 45 in which they were larger than the extent of surgery, there were 48 cases in which the PET abnormalities were comparable to the resection or disconnection regions.

For pathology, children with malformation of cortical development (MCD) accounted for the vast majority (87 cases, 77.0%; Table 2).

### 3.4 Complications

In terms of long‐term postoperative complications, in addition to new hemiparesis or hemianopia caused by hemispherotomy, which was predicted before surgery, one child underwent a ventriculoperitoneal (VP) shunt procedure 8 months after undergoing a temporal lobectomy, one suffered hydrocephalus without any process, and five patients had weakness in the contralateral extremity. There were no deaths or other long‐term complications. Early postoperative complications included transient nonbacterial inflammatory reactions manifesting as a short‐term fever in 60 (53.1%) patients, which was fully resolved before discharge from the hospital, and three patients who suffered from bacterial inflammation were cured by antibacterial treatment before discharge. Seven children manifested oral twitching or unilateral limb clonic episodes due to cerebral edema, which resolved within 3–7 days after dehydration therapy.

### 3.5 Seizure and development outcome

**FIGURE 3:**
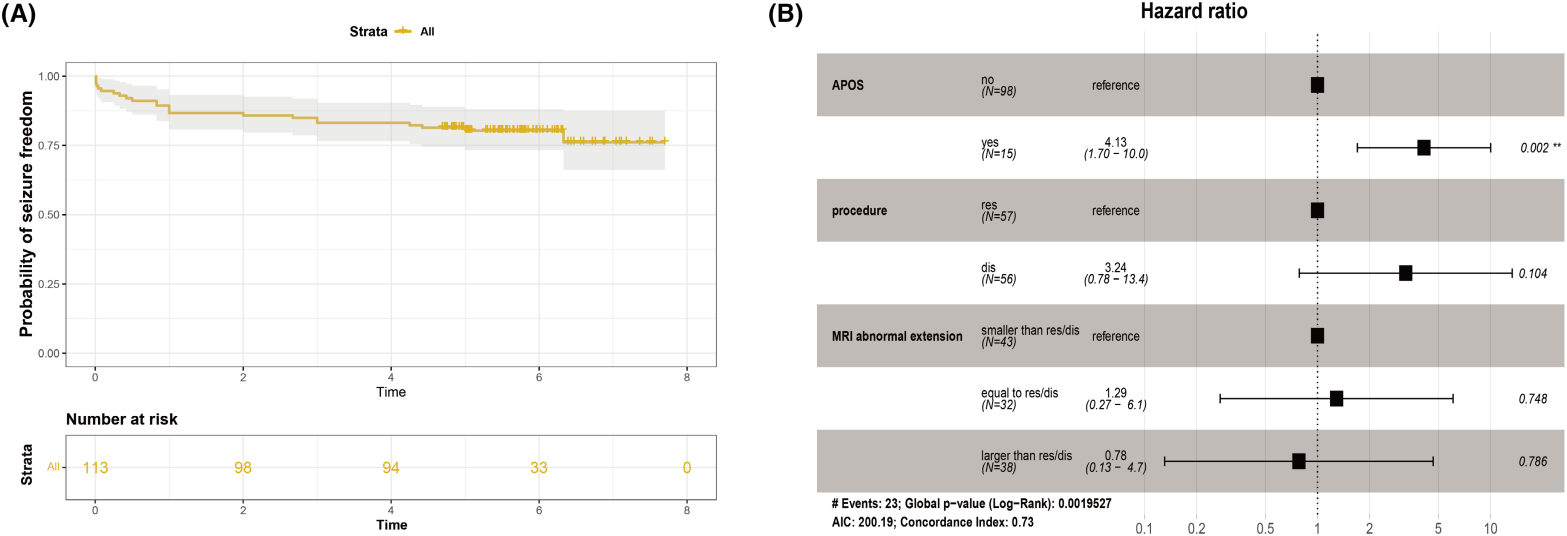
(A) Survival analysis illustrating the chances of postsurgical seizure freedom; (B) multivariable Cox analysis of APOS (*p* = 0.002, HR = 4.13), surgical procedures (*p* = 0.104, HR = 3.24), MRI abnormal extension equal to res/dis (*p* = 0.748, HR = 1.29), and larger than res/dis (*p* = 0.786, HR = 0.78). APOS occurrence was more likely to predict seizure recurrence after correcting for two additional factor.

At the last follow‐up, 98 patients (86.7%) were free from seizures. Among the patients who experienced recurrence, there were six Engel II cases, one Engel III case, and eight Engel IV cases. One year after surgery, the rate of seizure freedom reached 86.7% (98/113), which was as high as the last follow‐up (Figure 3A). All four patients who had definite pathogenic genes were seizure‐free. Fifteen patients underwent more than one surgery after the initial curative surgery, including four patients who underwent two more surgeries. Vagus nerve stimulation was conducted as a palliative therapy in four patients, which improved seizure outcomes to some extent. Ten of the patients who underwent multiple operations were seizure‐free at the last follow‐up (Table 3).

**TABLE 3:** Repeated procedures.

	Sex	Age	Side	Initial surgery	Second surgery	Third surgery
1	Male	2.30	R	Parietal resection	Enlargement resection	VNS
2^a^	Female	1.58	R	Central cortex resection	Enlargement resection	
3	Male	2.48	R	Hemispherotomy	VNS	
4	Female	1.10	L	Hemispherotomy	Re‐hemispherotomy	
5	Female	2.39	R	TPO disconnection	VNS	Frontal disconnection
6^a^	Female	1.23	R	Frontal resection	Enlargement resection	
7^a^	Male	2.72	L	Frontal and insular resection	Enlargement resection	
8^a^	Male	1.18	L	Hemispherotomy	Re‐hemispherotomy	
9^a^	Male	0.84	R	Hemispherotomy	Re‐hemispherotomy	
10^a^	Female	1.36	L	Frontal resection	Subtotal hemispherotomy	
11^a^	Male	2.38	R	Central cortex resection	Enlargement resection	
12^a^	Male	1.79	L	Frontal resection	SEEG	Enlargement resection
13	Male	1.15	L	TPO disconnection	Enlargement resection	VNS
14^a^	Female	1.24	L	Hemispherotomy	Re‐hemispherotomy	
15^a^	Female	2.05	R	Frontal and insular resection	Enlargement resection	

^a^ Seizure‐free at the last follow‐up.

The nonparametric independent‐sample Kruskal–Wallis test revealed that the distribution of Engel classification was associated with APOS occurrence (*p* < 0.001), MRI abnormality compared to resection/disconnection (*p* < 0.001) and surgery procedure types (*p* = 0.002). These three factors were tested in a multivariate Cox analysis of seizure outcome (Figure 3B). The multivariate Cox analysis showed that APOS (HR = 4.13, 95% CI: 1.7–10.0, *p* = 0.002) occurrence was more likely to predict seizure recurrence after correcting for two additional factors. In addition, 13 of 15 children who experienced recurrent seizures had pathologically confirmed MCDs. However, the statistical results of the correlation between pathology and prognosis did not show a significant difference.

The postoperative follow‐up time ranged from 45.7 to 89.1 months, with an average of 61.15 months. Medication was discontinued at various times in 90 (79.6%) patients postoperatively, with ASM therapy being completely discontinued in 36 (31.9%) children. Investigating the postsurgical EEG of 15 patients with recurrent seizures, seven patients had ipsilateral interictal or ictal discharge, while seven patients had mostly abnormal EEGs in the contralateral hemisphere beyond the surgical area. One patient had no EEG examination after surgery.

**FIGURE 4:**
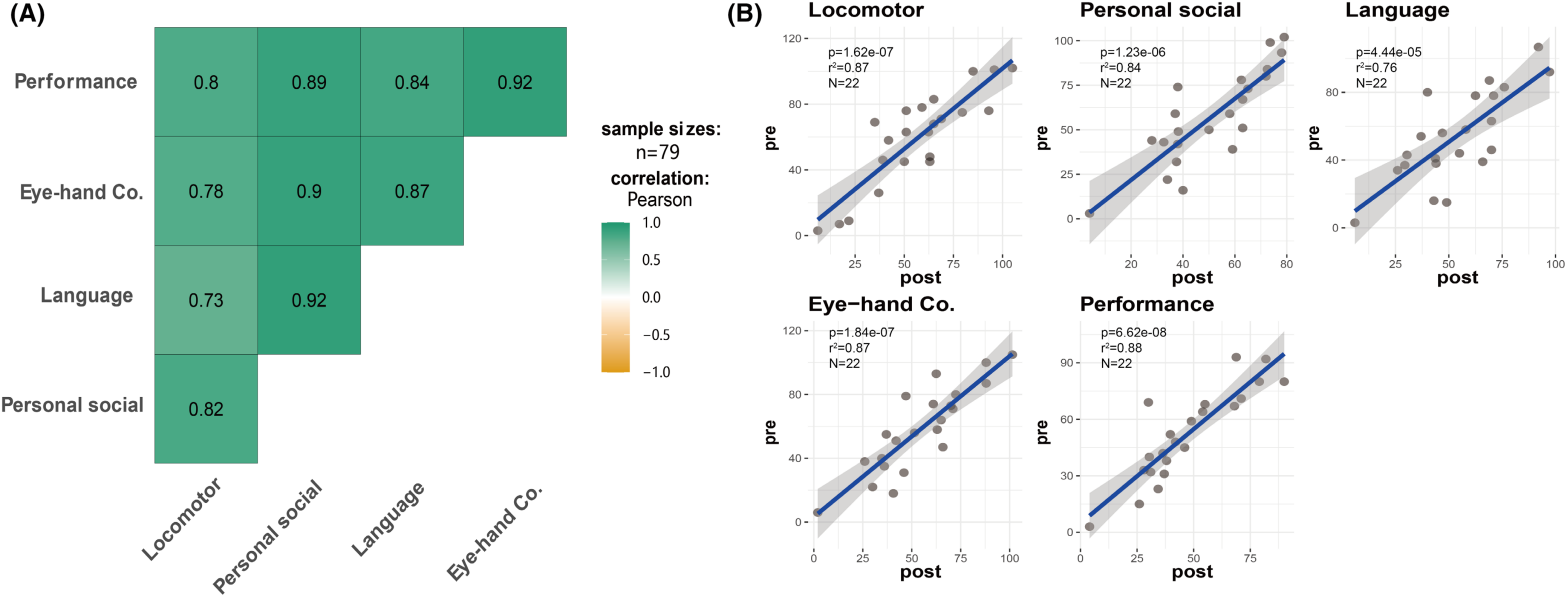
(A) Griffith DQ domain correlation using the Pearson test showing high two‐by‐two correlation in the subscale; (B) Griffith DQ high correlation between the pre‐ and postoperative periods using the Pearson test (*p* < 0.001).

**FIGURE 5:**
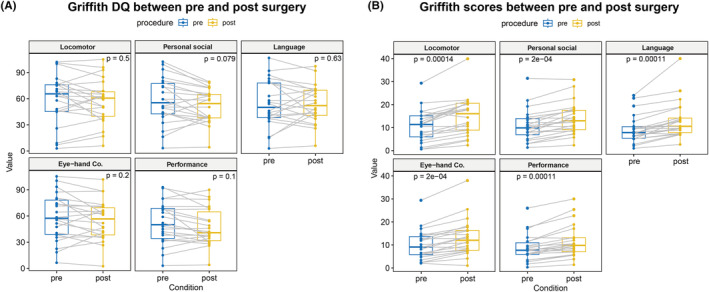
Griffith DQ correlation between the pre‐ and postoperative periods using paired *t*‐tests.

Preoperative GMDS‐C DQ correlation analysis of 79 cases showed significant correlations between any pairwise comparison of the five domains (motor, individual‐social, language, hand‐eye coordination, and performance; *p* < 0.05; Figure 4A). In 22 patients with available pre‐ and postoperative cognitive assessments, the correlation was significant in each domain (*p* < 0.01; Figure 4B). Despite noticeable improvement in the raw scores (*p* < 0.01), there was no significant difference in the pre‐ and postoperative results of the GMDS‐C or the DQs (*p* > 0.05; Figure 5).

## 4 DISCUSSION

It has been reported that the seizure‐free rate in children who are younger than 3 years of age who undergo surgical treatment for DRE ranges from 61.5% to 80%.^18^ In our study, we retrospectively analyzed the postsurgical seizure prognosis and development of 113 children who were younger than 3 years of age. At the last follow‐up, the seizure‐free rate was 86.7% with a follow‐up duration of at least 3 years. The seizure‐free rate at 1 year postoperatively was the same as that at the last follow‐up. Children with recurrent epilepsy mostly had seizures within 1 year after surgery, most frequently occurring within 3 months postoperatively.^17^ The number of ASMs taken was reduced to various extents in 79.6% of patients, with 31.9% being completely taken off the ASMs postoperatively. Children under 3 years old can have long‐term stable and satisfactory seizure outcomes with surgical treatment.

Most studies in the literature have focused on analyzing the surgical prognosis and predictors, but there have been fewer reports on the strategies for preoperative assessment, including how to determine the boundaries of the epileptogenic lesion during surgery and the best surgical approach to obtain optimal seizure and developmental outcomes. It is well known that the preoperative evaluation is highly related to the surgical prognosis for DRE and the key to the success is complete removal of the epileptogenic zone (EZ).^17^ Several publications have reported that residual epileptogenic lesions after surgery are highly likely to cause postoperative interictal discharges, thereby causing recurrent seizures.^19^, ^20^ However, the EZ has not been clearly defined in our clinical work. There is no absolute biomarker to determine the location and extent of the EZ. Because the clinical characteristics of DRE in children under 3 years old differ greatly from those of older children and adults, the preoperative evaluation and surgical approach must also differ, especially with respect to anatomical‐electrical‐clinical correlations.^21^

### 4.1 Localization of the EZ in children under 3 years old

In our center, the location of the EZ is first found based on the findings of the standard 3D MRI sequence. Both an EEG and a semiology assessment play an auxiliary role in the localization of the EZ. In principle, anatomical lesions are considered the most critical factors even if the anatomical‐electrical‐clinical information is not entirely concordant with each other.^22^ In such cases, the “lesion‐dominant” rationale might be considered a priority. In our cohorts, 100% of patients had structural lesions on MRI. Surgical resection/disconnection of areas that are equal to or larger than abnormalities found on MRI is essential to obtain optimal epilepsy outcomes.^20^ Most of the patients in our cohort had MCDs, which have been recognized as being highly epileptogenic. Oftentimes, the lesion can only be recognized by very experienced epileptologists or epilepsy surgeons, especially for very young children, so finding the MCD is significantly conducive to identifying the EZ. In such circumstances, multifocal or generalized interictal epileptic discharges on scalp EEG are not a contraindication for surgery. For example, 66 percent of children in our cohort suffered from spasms, so it was impossible to localize the EZ via scalp EEG or semiology.^23^ In contrast, if there is absolutely no structural lesion on MRI in a young epilepsy patient, even if there are symptoms and focal evidence on the scalp EEG, conservative treatments, such as a ketogenic diet or vagus nerve stimulation, should be suggested first. Some underlying potential etiologies, such as genetic mutations, could not be excluded. Overall, lesions on MRI are the most reliable factors that can be used to identify the EZ, thus contributing to the success of surgical treatment.^23^, ^24^

In our center, the strategy for localizing the EZ in children under 3 years old is to consider MRI abnormalities as the first criterion. Structural lesions that are confirmed by consulting electroclinical data are an indication for curative surgery. Genetic testing is usually required for very young children, especially those with large MCDs. In regard to repeated procedures, seven children with recurrent seizures were cured by enlarged resection around the first resection area or in other lobes. This further demonstrated the great value of complete removal of the epileptogenic lesion. Nowadays in our center, if a patient has multiple MCDs or other lesions in unilateral hemisphere, or the motor cortex is involved in the lesion, a “staged procedure” could be carried out before careful discussion with the family. The first stage of surgery is performed according to the patient's anatomical‐electrical‐clinical relationship with the motor cortex spared. If seizures recur after the first surgery, the remaining lesions should be resected completely in the second stage.

### 4.2 Determining the extent of surgical resection

**FIGURE 6:**
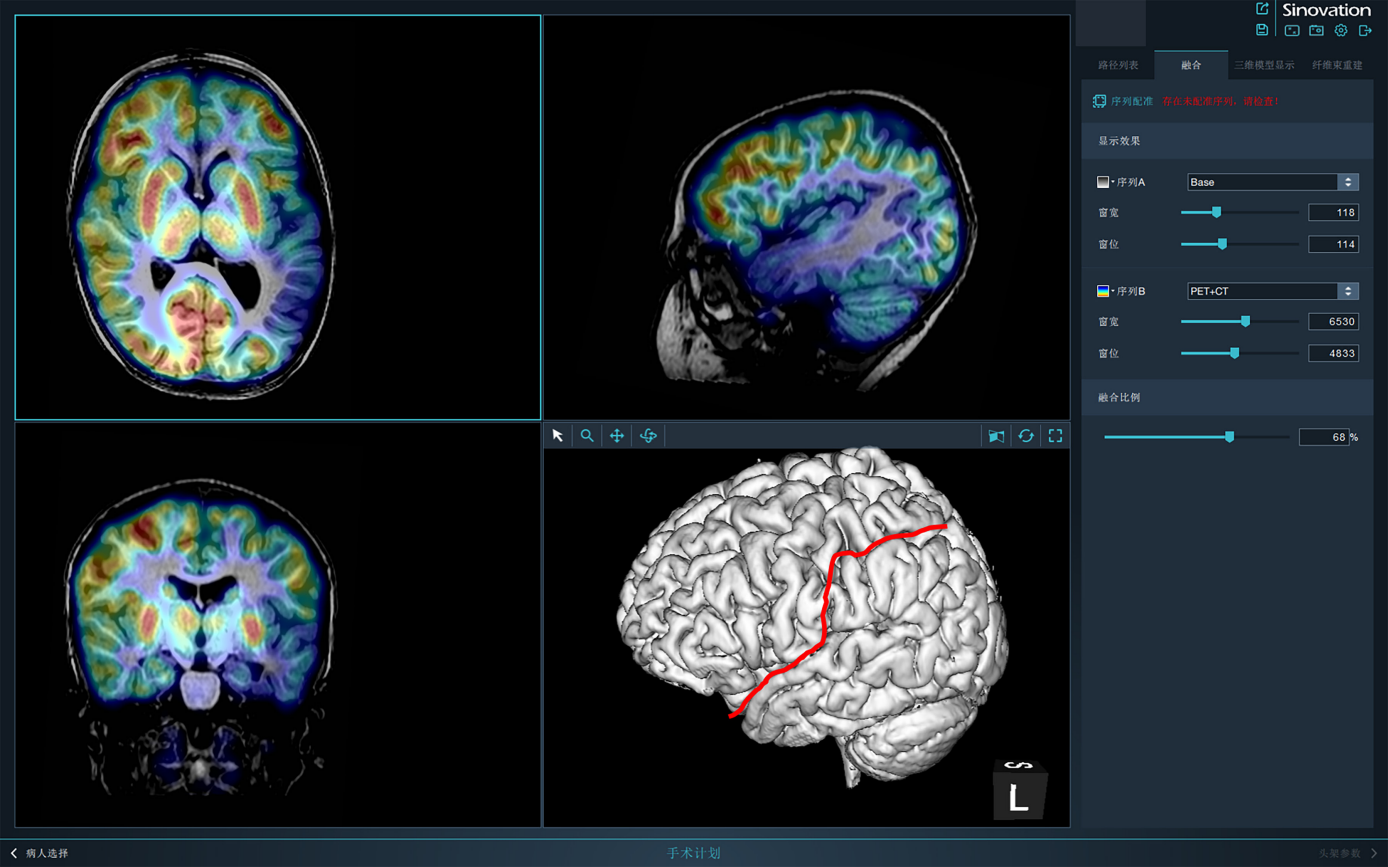
Temporal–parietal–occipital disconnection surgery plan. Imaging of T2 flair, MRI‐PET fusion, and 3D brain reconstruction. Sinovation software illustrated MRI T2 flair of axial and sagittal view of one patient with MCD showing thickening of gray matter and blurred white–gray matter boundary in left occipital lobe, posterior parietal, and temporal lobe. MRI‐PET fusion indicated that the low metabolism lesion was more obviously in the right posterior cortex. This software was used for a 3D brain reconstruction and aided in the decision of the resection range after discussion.

No patients in this cohort underwent an invasive intracranial EEG. In young children, even if many electrodes are implanted, the interictal discharges are usually quite extensive, and the immature epileptogenic network is difficult to specify.^25^ Because the white matter of the brain in children under 3 years is poorly myelinated and their networks are not yet established,^26^ precise localization of the EZ with intracranial electrodes is difficult to achieve. However, successful determination of the precise extent of both anatomical and epileptogenic lesions is the key to achieving seizure‐free outcomes. In our center, both MRI and PET imaging data are carefully evaluated simultaneously with the assistance of Sinovision software, and then, a 3D brain model of the epileptogenic lesion is fused. With the above information, the boundaries of the EZ can be easily decided. Furthermore, the surgery is planned with the consideration of all the other presurgical information, especially the scalp EEG (Figure 6).

Here, we summarize the steps for deciding extent of the final resection: (1) The extent of the lesion on MRI is judged layer by layer on the software, and the boundaries are interpreted by the neurosurgeons with careful analysis of each sequence. (2) Coregistration of MRI with PET images by Sinovision software is mandatory for further decisions regarding surgery extension. In most cases, the extent of hypometabolism on PET is more extensive than the extent of the lesion on MRI,^27^ and this information must be considered very seriously when deciding the extent of surgery. (3) During resection in the OR (operation room), the neurosurgeon can slightly modify the surgical boundary according to both the sensation of abnormal brain tissue and the result of the ECoG. This may improve the surgical outcome if neuroimaging fails to reveal the real border of the potential lesion. (4) Lesions involving the cortex should be managed carefully. Resection of the anatomical structure of the central cortex can be performed only when the lesion is confirmed via MRI. Surgery can be safely performed in children under 3 years old with lesions in the language cortex. In such circumstances, the anatomy of the language cortex is not capable of developing any language function in the same hemisphere. Even if there is partial ipsilateral language development, it can be compensated for by strong neuronal plasticity of the brain in the cortices of other areas.^28^, ^29^

### 4.3 The selection of optimal surgical procedures and the prevention of surgical complications

Only one patient underwent VP shunt treatment due to postoperative hydrocephalus. The lower incidence might be caused by the following reasons: (1) there were more children with MCD than patients with encephalomalacia in our cohort. Patients with encephalomalacia had a significantly higher probability of postoperative hydrocephalus than those with MCD based on the literature.^30^ The high incidence of postoperative hydrocephalus after anatomic hemispherectomy is similar to patients with very severe hemispheric encephalomalacia. (2) Approximately 50% of the dissecting surgeries in this study utilized a disconnection surgical approach, preserving the vast majority of the brain tissue within the cranial cavity, which was very important for cerebrospinal fluid circulation, and reduced the incidence of hydrocephalus. (3) In hemispherotomy, since the hemisphere ventricular system was essentially completely open, we routinely placed a subdural drain for at least 1 week and no more than 2 weeks until the cerebrospinal fluid became clear in color. We believe this procedure is very effective in preventing postoperative obstruction of cerebrospinal fluid flow.

Disconnection surgeries were performed in approximately half of the patients (56, 49.5%). In all disconnection surgeries, 18 children had nonhemispheric lesions, including 14 TPO disconnections, 1 frontal–parietal lobe disconnection, 1 temporal–occipital lobe disconnection, 1 parietal–occipital lobe disconnection, and 1 temporal lobe disconnection. In our center, the main indication for a disconnection approach is that, on MRI, the lesion or EZ appears to involve one or more lobes. Disconnection surgery is not indicated for the treatment of developmental tumors, such as ganglioglioma or dysembryoplastic neuroepithelial tumors, because of the increased possibility of tumor recurrence. Disconnection surgery is advantageous for young children because the extent and time of the craniotomy is small and short, the total operative time is short, the blood loss volume is low, and the EZ can be completely resected. In addition, disconnection procedures can significantly reduce the incidence of postoperative complications, such as hydrocephalus, subdural effusion, and infections.^31^ However, the disconnection surgery technique requires that neurosurgeons have more surgical skill and experience; otherwise, incomplete disconnection of the epileptogenic lobe increases the possibility of epilepsy surgery failure.

The youngest child in our cohort was 3 months old and weighed 5 kg. Patients who are of a younger age or a lighter weight are more likely to experience life‐threatening risks and often do not undergo presurgical evaluations or surgical treatment due to lack of sufficient presurgical observing time. Masaki Iwasaki and his colleagues followed the same criteria in selecting the best surgical candidates.^32^ Therefore, in our center, unless it is a life‐saving emergency, curative surgery is only indicated for children who are older than 3 months of age and weigh more than 5 kg.

### 4.4 Prognostic factors

In the multivariate analysis, APOS was found to be the most crucial independent predictor associated with seizure recurrence, which was also shown in earlier studies.^11^, ^17^, ^33^ Early postoperative seizures are considered a key marker for surgical failure and can even predict future secondary surgery.^34^ Disconnection procedures are associated with postoperative seizure recurrence, which might be caused by a large EZ that could not be completely resected. On the contrary, when the epileptogenic lesion is very close to the motor cortex or the insula is involved, especially in MCD patients, complete resection of the EZ is very difficult. To date, we have not had a biomarker that could accurately predict difficult resection or SEEG implantation due to limitations in evaluating the child's clinical characteristics.^35^ The procedure mentioned above could solve the problem as much as possible.

Twelve of 15 patients with recurrent epilepsy had MCDs, which was the most common etiology in patients whose surgery failed. This result is consistent with previous studies.^18^ However, MCD was not statistically associated with a higher rate of seizure recurrence. The reason for this is that most cases were MCDs.

### 4.5 Surgical influence on developmental assessment

The GMDS is a psychological developmental assessment scale for children aged 0–8 years. The GMDS‐C scale was revised according to the second edition of the GMDS 2006, which is more appropriate for Chinese children.^36^ Pre‐ and postoperative GMDS‐C scores were compared for each domain to assess the effect of surgery on neurological development. The raw scores were significantly different, but there was no difference in the DQs. A shorter seizure duration was associated with better DQs.^37^, ^38^ Patients with a high seizure frequency have low preoperative developmental scores. There were significant differences in the raw scores, indicating that epilepsy surgery had a positive impact on developmental outcomes. However, due to the differences of underlying etiologies and brain volume resection, shorter duration of postoperative GMDS‐C assessment and side effects of AEDs after the surgery, DQ showed an obvious decreasing trend when compared with normal children. Recent reports in the literature on surgery for medically refractory epilepsy under the age of 6 months or older children have also yielded results of elevated bare scores and decreased DQ on postoperative developmental assessments.^3^, ^39^ Our study also demonstrated that preoperative development is a major determinant of postoperative outcome, which was also demonstrated previously.^11^

## 5 CONCLUSIONS

For young children under 3 years old who have epilepsy caused by structural abnormalities, early curative treatment has good long‐term surgical seizure outcomes and a low complication rate. A good presurgical evaluation and a sensible resection approach are crucial. Our study has the following limitations: A small number of patients experienced postoperative seizure recurrence, which may have caused statistical bias, and a systematic evaluation of the cognitive prognoses of a larger group of patients over a longer period is needed.

## AUTHOR CONTRIBUTIONS

Lixin Cai, Xiaoyan Liu, and Yuwu Jiang designed this study and revised the manuscript. Hao Yu and Qingzhu Liu analyzed the data and drafted and revised the manuscript. Ruofan Wang collected the data. Yu Sun, Chang Liu, and Yao Wang helped to select the patients. Taoyun Ji and Shuang Wang helped to interpret the EEG data. Ruofan Wang followed the patients. All the authors contributed to the article and approved the submitted version.

## CONFLICT OF INTEREST STATEMENT

None of the authors have any conflicts of interest to disclose.

## Data Availability

The raw data supporting the conclusions of this article can be obtained from the authors upon reasonable request.
